# Development of an Improved Method for the Isolation and Culture of Newborn Sheep Primary Hepatocytes

**DOI:** 10.3390/cimb44080248

**Published:** 2022-08-12

**Authors:** Bowen Chen, Xiaoning Dou, Dan Zhang, Tiaoguo Liu, Bohui Yang, Zengkui Lu

**Affiliations:** 1 Key Laboratory of Animal Genetics and Breeding on Tibetan Plateau, Ministry of Agriculture and Rural Affairs, Lanzhou Institute of Husbandry and Pharmaceutical Sciences, Chinese Academy of Agricultural Sciences, Lanzhou 730050, China; 2 Sheep Breeding Engineering Technology Research Center of Chinese Academy of Agricultural Sciences, Lanzhou 730050, China

**Keywords:** sheep, primary hepatocytes, isolation, culture, identification, optimization

## Abstract

The liver plays a crucial role in metabolism, synthesis, biotransformation, secretion, and excretion. Hepatocytes are the main cells of the liver and can be used as a cell model to study liver function. The classic method of collagenase perfusion to isolate hepatocytes is a two-step technique that is time-consuming, labor-intensive, and has high technical requirements. Therefore, in this study, we compared different methods for isolating and culturing primary hepatocytes. We found that the 0.25% trypsin and 0.1 mg/mL type IV collagenase mixture at a 1:1 ratio showed the most efficient cell digestion, and William’s Medium E complete medium showed the best growth and proliferation. The isolated cells showed the typical irregular polygonal morphology of hepatocytes. Periodic acid–Schiff staining and immunofluorescence confirmed that the isolated cells were positive for glycogen and hepatocyte-specific markers cytokeratin 18, AFP, and albumin. On subculturing, stable cell lines were obtained. Therefore, we optimized the isolation and in vitro culture method to obtain highly pure (>95%) sheep primary hepatocytes from newborn sheep liver tissue.

## 1. Introduction

The liver plays a vital role in metabolism. Hepatocytes in make up 80% of the liver mass [1]. Hepatocytes have enormous (and perhaps unlimited) replication potential [2,3]; however, the proliferation of isolated primary hepatocytes is hardly in vitro [4]. Therefore, obtaining stable hepatocytes is difficult. Primary hepatocytes make an ideal model for studying nutritional and hormonal regulation of ruminant liver metabolism [5]; however, sheep hepatocytes have been isolated and cultured with limited success [6,7]. Furthermore, there exists no viable in vitro cellular model of primary hepatocytes due to the difficulty in isolation.

Currently, the two-step collagenase perfusion protocol is the most used classic method for hepatocyte isolation [3,8,9,10,11]. Berry and Friend first described this technique in 1969 for the isolation of rat hepatocytes [12]. The procedure includes anesthesia, cannulating the inferior vena cava, perfusing calcium chelators to remove blood and calcium ions, perfusing a large amount of collagenase, and filtering the tissues through fine sieves to obtain liver cells [10,13]. However, the procedure has significant drawbacks, including the need for at least 2 h of exposure to hepatocytes obtained from these procedures, the use of a large amount of collagenase, and excellent operating technology. This method is labor-intensive, time-consuming, and requires high technical skill [10]. Therefore, a simple and efficient method to isolate hepatocytes is urgently needed.

In this study, we propose an improved and optimized method for the isolation and characterization of primary hepatocytes from newborn sheep liver tissue. We used five different methods to optimize hepatocyte isolation conditions, and we also investigated the best medium for culture for high proliferation. The isolated hepatocytes were characterized by periodic acid–Schiff (PAS) staining for glycogen and by alpha-foetoprotein (AFP) expression as well as the hepatocyte-specific markers cytokeratin 18 and albumin. Additionally, we subcultured hepatocytes in vitro and obtained stable and sustainable cells to lay the groundwork for future studies of hepatocytes and livers.

## 2. Materials and Methods

### 2.1. Media, Reagents, and Chemicals

(1) Complete medium: William’s Medium E (Gibco, Carlsbad, CA, USA. cat. no. 2318299) containing 15% FBS (Gibco, Carlsbad, CA, USA. cat. no. 10099141C) and 2 mM L-glutamine (Gibco, Carlsbad, CA, USA. cat. no. 25030081, 200 mM) prepared as per the manufacturer’s instructions.

(2) Type IV collagenase (Sigma-Aldrich, St. Louis, MO, USA. cat. no. C5138).

Collagenase stock solution was prepared by dissolving 20 mg of type IV collagenase in 20 mL of PBS (1X, pH 7.2–7.4, 0.01M) (Solarbio, Beijing, China, cat. no. P1020) to prepare a 1 mg/mL collagenase stock solution. The stock solution was filter sterilized using a 0.22 µm filter and stored at −20 °C until further use.

For cell dissociation, we prepared a solution by adding 5 mL of collagenase stock solution to 45 mL of PBS (1X, pH 7.2–7.4, 0.01M) for a final concentration of 0.1 mg/mL.

(3) Rat tail collagen-coated plates.

Rat tail collagen stock solution was prepared by dissolving 10 mg of rat tail collagen in 10 mL of acetic acid (0.1M) to 1 mg/mL stock solution. This solution was stirred continuously at room temperature for at least 1–3 h for complete dissolution, filter sterilized using a 0.22 µm filter, dispensed into 1 mL/tube, and stored at −20 °C. Rat tail collagen working solution was then prepared by adding 1 mL of the stock solution to 9 mL of PBS (1X, pH 7.2–7.4, 0.01M) to yield 0.1 mg/mL solution and thoroughly mixed.

Plate coating: 800 μL of the diluent was added to each well (8–10 μg/cm^2^) of a 6-well plate (costar, cat. no. 3516) and incubated overnight at 37 °C and then washed twice with 1 × PBS before use.

### 2.2. Sheep Liver Tissues Collection

Three 1-day-old newborn sheep (Hu sheep, 1.5–3 kg, ♂) from Lanzhou Wanshan Plantation and Breeding Professional Cooperative were used in this study. Those sheep were anesthetized with isoflurane inhalation (Sigma-Aldrich, St. Louis, MO, USA. cat. no. 792632) and then fixed on a trough-shaped stool, and the carotid artery was cut off for bloodletting and slaughter. After the bloodletting was completed, a sterile sharp knife was used to resect the sheepskin along the midline of the abdomen, the livers were obtained and immersed in normal saline containing 2% Penicillin-Streptomycin (PS, Gibco, Carlsbad, CA, USA. cat. no. 15140163) and then brought to the laboratory.

### 2.3. Primary Hepatocyte Isolation and Culture Procedures

Firstly, the collected livers were quickly sterilized with 75% ethanol in a plastic petri dish for 1 min, and then immediately transferred into a new plastic petri dish containing PBS with 1% PS and then rinsed three times in a new plastic petri dish to disinfect and wash away surface impurities along with any excess blood. Then, sterile scissors and tweezers were used to cut 1 × 1 cm^3^ liver tissues, and the following five processing methods were used to obtain cell suspensions.

A: The liver tissue was put in a small glass bottle and was cut into 1-mm^3^ pieces using sterile scissors. Then, 5 mL of complete medium was added to the cells and the cells were filtered using a 100 μm sieve.

B: 1 mg/mL type IV collagenase digestion: To the 1 mm^3^ tissue pieces, 5 mL of 1 mg/mL type IV collagenase was added for digestion and incubated in a 37 °C water bath for 15 min with continuous shaking until the digestion was complete. After digestion, a complete medium was added to stop the digestion, and the cells were filtered using a 100 μm sieve.

C: 0.1 mg/mL type IV collagenase digestion: To the 1 mm^3^ tissue pieces, 5 mL of 0.1 mg/mL collagenase was added to further digest the tissue and incubated at 37 °C in a water bath for 15 min with continuous shaking until the digestion was complete. After the digestion, a complete medium was added to stop the digestion, and the cells were filtered using a 100 μm cell sieve.

D: 0.25% trypsin digestion: To the 1 mm^3^ tissue pieces, 5 mL 0.25% trypsin (Gibco, Carlsbad, CA, USA, cat. no.15050065) was added for digestion, and incubated at 37 °C in a water bath for 15 min with continuously shaking until the digestion was complete. After the digestion, a complete medium was added to stop the digestion, and the cells were filtered using a 100 μm sieve.

E: 0.25% trypsin + 0.1 mg/mL type IV collagenase digestion: To the 1 mm^3^ tissue pieces, 5 mL 0.25% trypsin and 0.1 mg/mL type IV collagenase in a ratio of 1:1 were added for digestion and incubated at 37 °C in a water bath for 15 min with continuous shaking until the digestion was complete. After the digestion, a complete medium was added to stop the digestion, and the cells were filtered using a 100 μm sieve.

The cell suspension was centrifuged at 800 rpm for 5 min, the supernatant was discarded, resuspended in 3 mL ACK Lysis buffer (Solarbio, Beijing, China, cat. no. R1010), and incubated at room temperature for 10 min. The cells were again centrifuged at 800 rpm for 5 min, the supernatant was discarded and resuspended in a complete medium. Thereafter, the cells were cultured in a 6-well plate (5 × 10^5^ cells/cm^2^) coated with rat tail collagen at 37 °C in a 5% CO_2_ incubator for 2 days.

Finally, the non-adherent hepatocytes and dead cells were discarded the next day and 2 mL of new complete medium was added to the 6-well plate coated with rat tail collagen and incubated at 37 °C in a 5% CO_2_ incubator.

### 2.4. Cell Passage Cultivation

The cultured cells were washed with PBS once, digested with 0.25% trypsin-EDTA at 37 °C for 2 min, and then 1 mL of complete medium was added to stop the digestion. The cells were then centrifuged at 1000 rpm for 5 min, resuspended in a complete medium, and inoculated into 6-well cell culture plates at 37 °C with 5% CO_2_ incubator.

### 2.5. Cell Glycogen Periodic Acid Schiff (PAS) Staining

Cell glycogen was stained by PAS as per manufacturer instructions (Solarbio, Beijing, China, cat. no. G1281). Briefly, the well plate with hepatocytes was washed once with 1 × PBS and 4% paraformaldehyde (Sigma-Aldrich, St. Louis, MO, USA. cat. no.158127) was added to the well plate, and cells were fixed at room temperature for 30 min. Then the plate was washed twice with distilled water and oxidizing agent was added at room temperature for 5–8 min (negative controls did not go through this step). The plate was then washed twice with distilled water. The sample was added to 1 mL Schiff Reagent and placed in the dark at room temperature for 10–20 min. Finally, the sample was differentiated in acidic differentiation solution for 2–5 s, and rinsed with distilled water. Then, 1 × PBS was added, and cells were observed under a Leica DMI6000B with adaptive focus control microscope (Wetzlar, Germany, HCX PL FLUOTAR 40x/0.6) and the images were processed with Leica LAS AF software.

### 2.6. Cell Proliferation Detection

The CCK-8 (Cell Counting Kit-8, ZETA LIFE, Menlo Park, CA, USA, cat. no. C7661) was used for monitoring cell proliferation as per the manufacturer’s instructions. Approximately 2 × 10^−3^ cells were seeded into a 96-well plate, CCK-8 (10 μL) was added to each well and the cells were incubation at 37 °C for 2 h. The optical density (OD) was then measured using a Multiskan FC-type microplate reader at 450 nm. Every group was repeated six times.

### 2.7. Immunofluorescence Assay

The cultured cells were washed once with PBS, and then fixed in 4% paraformaldehyde for 15 min at room temperature and washed with PBST ((PBS containing 0.25% Tween-20 (Sigma-Aldrich, St. Louis, MO, USA, cat. no. P9416)) three times for 5 min. Then, the cells were permeabilized with PBS containing 0.3% TritonX-100 (Sigma-Aldrich, St. Louis, MO, USA, cat. no. T8787) for 10 min at room temperature and washed with PBST three times for 5 min each. The cells were incubated in the blocking solution for 1 h with PBS containing 5% FBS and 0.3% TritonX-100. Thereafter, the cells were incubated with cytokeratin 18 antibody (Proteintech, Rosemont, IL, USA, cat. no. 10830-1-AP), AFP antibody (Alpha-fetoprotein, Proteintech, Rosemont, IL, USA, cat. no. 14550-1-AP), and albumin antibody (Proteintech, Rosemont, IL, USA, cat. no. 66051-1-Ig) overnight at 4 °C in 500 μL of antibody dilution buffer ((containing 0.3% TritonX-100 and 1% Bovine serum albumin (Sigma-Aldrich, St. Louis, MO, USA, cat. no. A1933)) with the antibody. Thereafter, the antibody-labeled cells were subsequently stained with the goat anti-rabbit IgG conjugated with Alexa Fluor^®^ 564 (Invitrogen, Waltham, MA, USA, cat. no. 2129899) or goat anti-mouse IgG conjugated with Alexa Fluor^®^488 (Gibco, Carlsbad, CA, USA, cat. no. 31160) in 500 μL of antibody dilution buffer for 1 h at room temperature. The cells were rinsed with PBST and incubated with DAPI (Solarbio, Beijing, China. cat. no. C0065) for 5 min at room temperature, then imaged using a ZEISS LSM800 confocal laser scanning microcopy (Munich, Germany, Plan APOCHROMAT 10x/0.45) and the image processing was carried out with ZEN software.

### 2.8. Statistical Analysis

Statistical analyses were performed using the GraphPad Prism 8 (San Diego, CA, USA, www.graphpad.com). Quantitative data were presented as the mean ± SEM. The level of significance was calculated using a one-way analysis of variance (ANOVA) and Tukey’s post hoc test. *p* < 0.05 was considered significant.

## 3. Results

### 3.1. Isolation and Culture of Primary Hepatocytes

**Figure 1:**
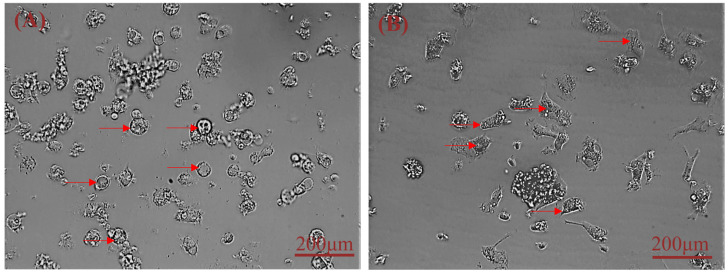
Morphological characteristics of isolated primary sheep hepatocytes (brightfield imaging). (**A**) Hepatocytes adhered to the surface after 2 days. (**B**) At low density, hepatocytes acquire an irregular polygon shape after 4 days. Note: red arrows indicate primary hepatocytes.

Freshly isolated primary hepatocytes are transparent and round. We found that cells adhered to the plate after 1 day of culturing and were spherical. After 2 days of culture, most of the hepatocytes adhere to the plate, they transformed from spherical to flat polyhedral with abundant cytoplasm and clear nuclei and appear sparsely plated (Figure 1A). Within 4 days, 85% cells displayed an irregular polygon and began to proliferate (Figure 1B).

**Figure 2:**
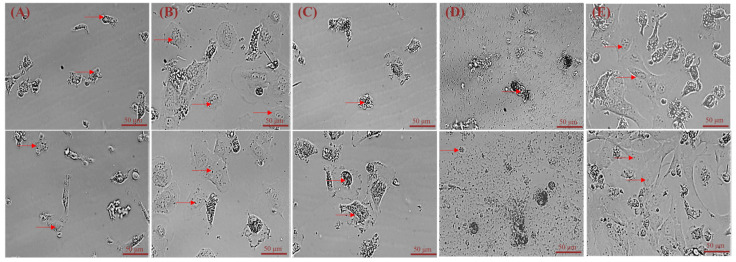
Morphological characteristics of primary sheep hepatocytes isolated using five different methods (brightfield imaging). (**A**) Liver tissue cut into 1 mm^3^ pieces; (**B**) Cut tissues treated subjected to 1 mg/mL type IV collagenase digestion; (**C**) Cut tissues treated subjected to 0.1 mg/mL type IV collagenase digestion; (**D**) Cut tissues treated subjected to 0.25% trypsin digestion; (**E**) Cut tissues treated subjected to digestion using a 1:1 0.25% trypsin +0.1 mg/mL type IV collagenase mix. The above image shows 4 days of culturing; the below image shows 6 days of culturing. Note: red arrows indicate primary hepatocytes.

The number of isolated primary hepatocytes by 5 different methods respectively were: 5.2 × 10^6^, 1.3 × 10^7^, 3.6 × 10^6^, 1.6 × 10^6^, and 3.3 × 10^7^. After 6 days of culture, all five digestion methods could yield different numbers of adherent primary hepatocytes (Figure 2A–E). The best digestion was achieved with the 0.25% trypsin and 0.1 mg/mL type IV collagenase 1:1 mixture, which led to the greatest number of adhered cells (Figure 2E).

### 3.2. Identification of Primary Hepatocytes

**Figure 3:**
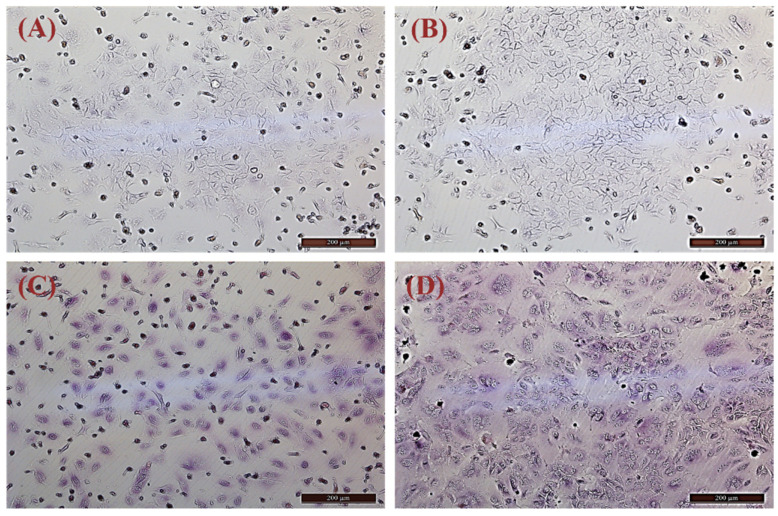
Primary hepatocytes subjected to PAS staining for glycogen (brightfield imaging). (**A**,**B**) Negative controls; (**C**,**D**) PAS staining of isolated cells.

The isolated primary hepatocytes were subjected to PAS staining for glycogen (Figure 3A–D). When compared with the negative control group, primary hepatocytes displayed purple-red or purple cytoplasm containing dense glycogen granules. Therefore, PAS staining revealed that the isolated cells were primary hepatocytes (cultured 6 days, passage 0).

**Figure 4:**
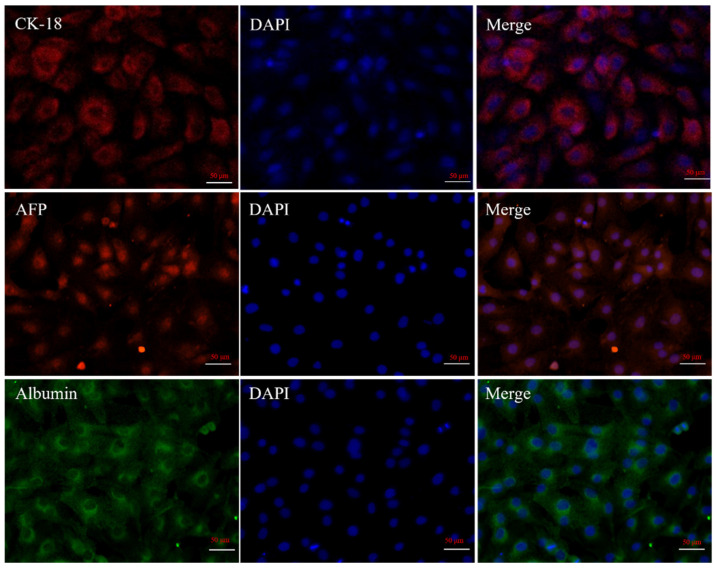
Characterization of primary sheep hepatocytes. Immunofluorescence analysis of isolated liver cells using antibodies corresponding to hepatocyte-specific marker cytokeratin 18, AFP, and albumin. Note: CK-18 and AFP are indicated with red fluorescence, albumin is green fluorescence, and DAPI is blue.

The isolated liver cells (cultured 6 days, passage 0) were assessed for hepatocyte-specific markers cytokeratin 18, AFP, and albumin using corresponding antibodies. Figure 4 shows that primary hepatocytes exhibited red or green fluorescence, and cell purity was at least 95% as determined by CK-18, AFP, and albumin staining.

### 3.3. Subculture of Primary Hepatocytes

**Figure 5:**
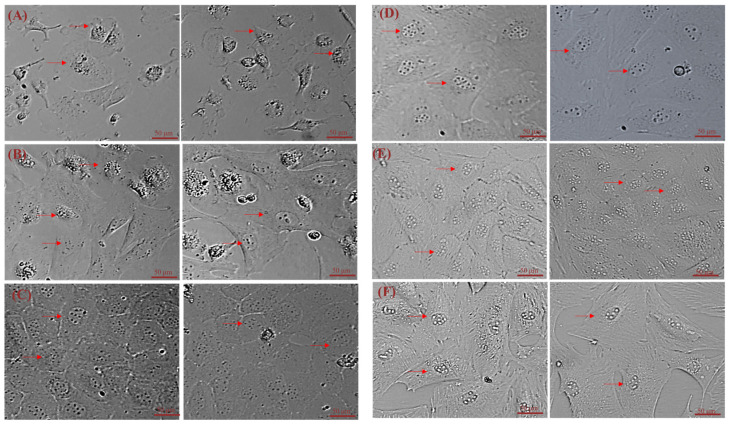
Steps of subculturing of primary sheep hepatocytes (brightfield imaging). (**A**) Passage 1; (**B**) Passage 2; (**C**) Passage 4; (**D**) Passage 7; (**E**) Passage 10; (**F**) Passage 5 after resuscitation. Note: red arrows indicate primary hepatocytes.

Primary hepatocytes grow fast. The growth of the cells to a density of 80–90% with the addition of 0.25% trypsin-EDTA was followed by the gradually shrinking of the cells to attain a spherical shape, which stopped digestion for the cells to be re-plated (passage at a ratio of 1:3). After their passage on the third day, they appeared as tightly connected irregular paving stones (Figure 5A). After reattachment, the cells covered almost the entire bottom surface. However, cells at a higher density were irregular polygons and were tightly connected while those at a lower density were larger polygons with evident nuclei. The cells were passaged every 2–3 days. Primary hepatocytes from newborn sheep can be passaged multiple times (Figure 5B–E).

During passage 2, cell proliferation was stable, and the morphological characteristics of hepatocytes were typical, demonstrating the capability for subculture and multiplication.

For preserving seed cells, after the subculture cells were digested and centrifuged, a freshly prepared cell cryopreservation solution (DMEM (Dulbecco’s modified eagle medium)/F12: FBS: DMSO = 2:1:1) was added. The cells were then placed in a cell cryopreservation box and frozen at −80 °C. Upon resuscitation (cryopreservation for 20 days), 70% of the cells were viable. Upon return to the state before cryopreservation, the cells displayed clear cell boundary after 1–2 days of culture (Figure 5F).

**Figure 6:**
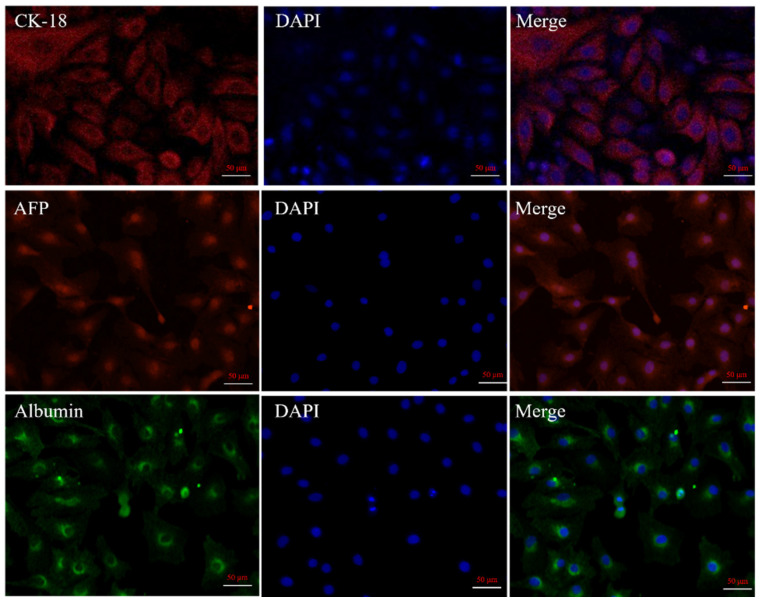
Characterization of resuscitated hepatocytes. Immunofluorescence analysis of resuscitated liver cells using antibodies corresponding to hepatocyte-specific marker cytokeratin 18, AFP, and albumin. Note: CK-18 and AFP are indicated with red fluorescence, albumin is green fluorescence, and DAPI is blue.

To further characterize the resuscitated hepatocytes, the cells were assessed for hepatocyte-specific markers cytokeratin 18, AFP, and albumin using corresponding antibodies. Figure 6 shows that resuscitated cells also exhibited red or green fluorescence, indicating the cells retained the characteristics of hepatocytes.

**Figure 7:**
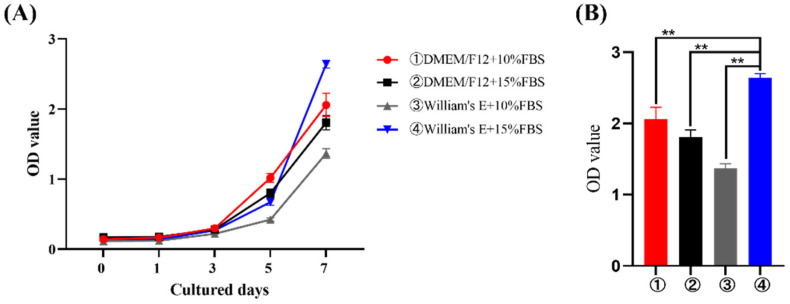
Proliferation assessment of sheep primary hepatocytes. (**A**) CCK-8 assay results of the proliferation of primary sheep hepatocytes cultured using four different culturing methods at days 0, 1, 3, 5, and 7. (**B**) Comparison of the OD of cultured cells on the 7th day. **, *p* < 0.01; ns, no significance.

After the CCK-8 assay, the OD increased with increasing culture time, suggesting that the cells continued to proliferate and that the fastest proliferation time was 5–7 days. The proliferation ability of primary hepatocytes was similar using the four different methods (Figure 7A). Furthermore, on day 7 of culturing, cells cultured in William’s Medium E containing 15% FBS had the highest OD value (Figure 7B), and were significantly different from the other three groups.

In conclusion, the results indicate that our proposed method can produce high-purity sheep hepatocytes that can be used to investigate the molecular mechanisms of liver function.

## 4. Discussion

The liver is the largest organ in animals and plays a role in complex processes, including metabolism, synthesis, biotransformation, secretion, and excretion. The liver has powerful detoxification functions, including flushing of both endogenous and exogenous toxins. Furthermore, the liver produces bile and secretes coagulation factors, as well as regulates blood volume, immunity, and regeneration. Primary hepatocytes are an excellent model for studying liver function.

Currently, two main methods are used to isolate primary hepatocytes. The first is in situ perfusion and collagenase digestion performed using occlusion of the ductus venosus and it results in high hepatocyte yield and purity. It is the classical and reliable method to obtain substantial primary hepatocytes, and has been used successfully for isolating human, mouse, rat, *Tupaia belangeri yaoshanensis*, calf, and fetal sheep liver cells [8,9,14,15,16,17]. Shen et al. applied the two-step collagenase perfusion method to isolate rat hepatocytes and achieved good results [8]. Mouse hepatocytes have also been isolated in Goldstein’s laboratory using this method [18]. Cao et al. isolated primary fetal sheep hepatocytes using perfusion solutions such as heparin sodium and collagenase [7]. However, this approach demands surgical techniques and is lengthy and cumbersome. A second approach involves breaking down the connective tissue network to release hepatocytes through mincing and enzymatic digestion. In their study, Xu et al. directly digested liver tissue with collagenase and obtained goose primary hepatocytes [19]. Zhao et al. prepared rainbow trout primary hepatocytes using 0.25% trypsin and 0.1% collagenase II in a 1:1 ratio [20]. In this study, we reduced collagenase concentration (0.1 mg/mL) and shortened digestion time based on these results. We compared the efficiency of five different methods to isolate hepatocytes by observing the adherence of liver cells (Figure 5A: 10%. 5B: 25%; 5C: 7%; 5D: 3%; 5E: 65%) and found that digestion with 0.25% trypsin and 0.1 mg/mL type IV collagenase mixture in a 1:1 ratio for 15 min showed the best results. We modified our cell isolation protocol to be simpler and less time-consuming while still achieving similar yield and purity. Microscopic observation of the isolated lever cells revealed that they were irregular polygons—a typical feature of hepatocytes. A large amount of hepatic glycogen is found in hepatocytes. Therefore, the purity of the isolated primary hepatocytes was confirmed by PAS staining for glycogen and immunofluorescence analysis (Figure 3 and Figure 4). PAS staining revealed that almost all cells were positive, confirming glycogen in the cytoplasm and thus, hepatocyte isolation. Moreover, immunofluorescence results revealed that the isolated cells expressed hepatocyte-specific markers such as cytokeratin 18, AFP, and albumin [7,21]. These results confirm that they were primary hepatocytes, and the culture method yielded a high purity. However, establishment and maintenance of hepatocyte polarity is essential for many functions of hepatocytes, therefore the identification of the phenotype of hepatocyte polarity and the assessment efflux by canalicular (apical) proteins require further study. In addition, this method of isolation of primary hepatocytes can be also applied to the other animal models [20], but the experimental conditions still need to be optimized, such as the concentration of type IV collagenase and the digestion time.

Primary fetal sheep hepatocytes are highly proliferative and can be subcultured for a long time. Researchers have found that rat embryonic hepatocytes gradually lose their epithelial phenotype when cultured in vitro, transforming into fibroblasts. Some studies have also shown that fibrotic features also appear after long-term culture of primary sheep hepatocytes [6]; a similar result was observed in this study when fetal sheep hepatocytes were cultured. Hepatocyte fibrosis can be caused by mechanical damage during the process of culture or passage. In liver fibrosis, nutrients and growth factors related to hepatocytes, such as hepatocyte growth factor (HGF)/epidermal growth factor (EGF) and insulin, can promote proliferation and repair [22].

Several methods for culturing primary hepatocytes have been reported [23]. Cao et al. used DMEM/F12 medium to culture fetal sheep hepatocytes [7,24,25]. Xu et al. used high glucose DMEM to culture primary goose hepatocytes [19,26]. Victoria et al. used William’s Medium E to culture human liver cells [17,27,28]. For determining the most suitable medium for the growth and proliferation of primary sheep hepatocytes, we used two types of medium and different concentrations of FBS and assessed cell proliferation. DMEM/F12 or William’s Medium E with 10–15% FBS sustained the normal proliferation ability of hepatocytes. However, William’s Medium E containing 15% FBS showed proliferation on the seventh day of culture. This indicated that primary sheep hepatocytes could be cultured best in William’s Medium E containing 15% FBS.

We also determined that the success of separating primary fetal sheep hepatocytes depends on the age of the sheep, the duration of isolation, the concentration of collagenase, the type of media, and the treatment of the cell culture plate. Firstly, the age of sheep plays an important role in the successful isolation of hepatocytes. Hepatocytes isolated from fetal or newborn sheep are the most suitable for culture because the adult liver exhibits very little proliferation [29,30]. Furthermore, it is crucial to isolate liver cells immediately after liver sample collection. The number of adherent hepatocytes reduced dramatically when the operation time was too long. Finally, collagenase concentrations must be appropriate to obtain proliferative hepatocytes. For obtaining adherent hepatocytes, 1 mg/mL collagenase can be used (Figure 2B), but we established that the 0.25% trypsin and 0.1 mg/mL type IV collagenase mixture is optimal. Rat tail collagen-coated dishes facilitate the adhesion of freshly isolated primary hepatocytes. Several studies have shown that primary hepatocytes do not require coating after passage and the cells can adhere to the walls quickly and proliferate, simplifying the experimental process.

In this experiment, we used mixed digestion of collagenase and trypsin to improve hepatocyte isolation and culture, and we successfully isolated and subculture stable hepatocytes from newborn sheep with good cell viability and high purity (>95%). The obtained sheep hepatocytes may be used in veterinary medicine to understand ruminant liver biology.

## 5. Conclusions

In this study, we propose an improved and optimized method for the isolation and characterization of primary hepatocytes from newborn sheep liver tissue. We tested five different methods to optimize the conditions for isolating primary sheep hepatocytes and found that hepatocytes adhered quickly to the collagen-coated plate when cultured in William’s Medium E with 15% FBS. In addition to PAS staining for glycogen and assessing AFP expression, the isolated hepatocytes were identified as hepatocyte using hepatocyte -specific markers such as cytokeratin 18 and albumin. We also subculture liver cells in vitro to obtain stable cells that may be valuable for future studies. The isolation of primary sheep hepatocytes (>95% purity) using our proposed novel method is expected to provide a near-infinite source of cells for multiple applications, as well as personalized drug screening and therapeutics in the future.

## Data Availability

Not applicable.
